# Reporter PET Images Bortezomib Treatment-Mediated Suppression of Cancer Cell Proteasome Activity

**DOI:** 10.1038/s41598-018-29642-w

**Published:** 2018-08-16

**Authors:** Jin Hee Lee, Kyung-Ho Jung, Cung Hoa Thien Quach, Jin Won Park, Seung Hwan Moon, Young Seok Cho, Kyung-Han Lee

**Affiliations:** 1 Department of Nuclear Medicine, Samsung Medical Center, Sungkyunkwan University School of Medicine, Seoul, Korea; 2 Department of Health Sciences and Technology, SAIHST, Sungkyunkwan University, Seoul, Korea

## Abstract

Proteasomal protein degradation is a promising target for cancer therapy. Here, we developed a positron emission tomography (PET) technique based on the sodium-iodide symporter (NIS) gene fused with the carboxyl-terminal of ornithine decarboxylase (cODC) that noninvasively images cancer cells with inhibited proteasome activity. A retroviral vector was constructed in which the murine cODC degron was fused to the human NIS gene (NIS-cODC). Transiently transduced CT26 and HT29 colon cancer cells and stably expressing CT26/NIS-cODC cells were prepared. In cancer cells transiently transduced with NIS-cODC, NIS expression and transport activity was low at baseline, but NIS protein and ^125^I uptake was significantly increased by inhibition of proteasome activity with bortezomib. Stable CT26/NIS-cODC cells also showed increased cytosolic and membrane NIS by bortezomib, and four different stable clones displayed bortezomib dose-dependent stimulation of ^125^I and ^99m^Tc-0_4_^−^ uptake. Importantly, bortezomib dose-dependently suppressed survival of CT26/NIS-cODC clones in a manner that closely correlated to the magnitudes of ^125^I and ^99m^Tc-0_4_^−^ uptake. CT26/NIS-cODC tumors of bortezomib-treated mice demonstrated greater ^124^I uptake on PET images and increased NIS expression on tissue staining compared to vehicle-injected animals. NIS-cODC PET imaging may allow noninvasive quantitative monitoring of proteasome activity in cancer cells treated with bortezomib.

## Introduction

Essential tumor-supporting machineries are an attractive target for cancer therapy^1^, and a key example is regulated protein degradation that occurs predominantly via the 26S proteasome complex^2,3^. Cancer cells characteristically have elevated proteasome activity^4^ because it offers a survival advantage by eliminating oncoproteins^5^. Indeed, treatment with proteasome inhibitors can induce cell cycle arrest and apoptotic death of cancer cells^6,7^. Therefore, the proteasome system is a promising target for cancer therapy and the ability to image its activity in living bodies could contribute to the development of new anticancer drugs.

An opportunity to identify cells with reduced proteasome activity is provided by specific protein sequences that are promptly recognized and eliminated through the proteasome system^8^. The C-terminal degron of mouse ornithine decarboxylase (cODC) is promptly recognized by 26S proteasomes for rapid ubiquitin-independent degradation^9^. Hence, in cancer cells, cODC-fused proteins undergo prompt degradation at baseline but accumulate when proteasome activity is suppressed by treatment with proteasome inhibitors. Vlashi *et al*. previously engineered cancer cells to stably express cODC-fused ZsGreen^10^, and cancer cells expressing this reporter were tractable in mice treated with proteasome inhibitors^11^. However, this technique has limited usefulness for quantitative monitoring of cancer cells in living bodies due to poor depth penetration of fluorescent signals.

The sodium iodide symporter (NIS) reporter can overcome this limitation and offers many additional advantages for *in vivo* imaging^12–14^. In human tissues, Expression of this selective iodide carrier is limited to the thyroid, salivary gland, gastric mucosa, and lactating mammary gland^15^. It does not influence underlying cell biochemistry, and by using species-specific sequences, it can avoid immune responses that are problematic with foreign reporter proteins. Furthermore, NIS imaging tracers do not require radiochemical synthesis, and multiple types of radioisotopes with a wide range of half-lives can be selected for positron emission tomography (PET) or γ-camera imaging. Indeed, our group has previously shown NIS gene imaging useful for tracking various types of cells in living bodies^13,14,16^.

In this study, we constructed a novel reporter system consisting of the human NIS gene fused to the cODC degron. Cancer cells transiently or stably transfected with the construct were assessed for NIS expression and substrate transport activity in response to proteasome inhibition. We further investigated the capacity of the NIS-cODC reporter to image tumors in mice treated with bortezomib with radioiodine PET.

## Results

### Proteasome inhibition of transduced cells increases NIS accumulation and substrate transport

**Figure 1:**
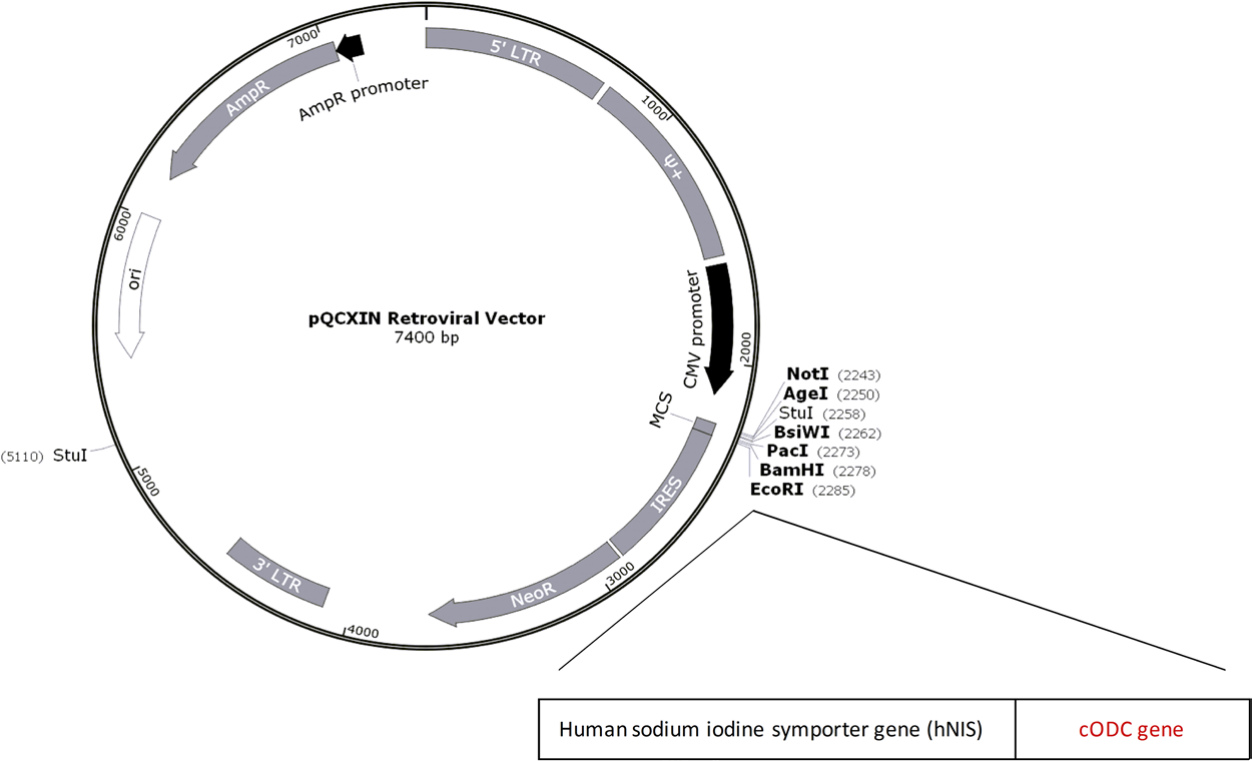
NIS-cODC construct. Illustration of pQCXIN retroviral expression vector containing the carboxyl terminal 37 amino acid sequence of the murine ornithine decarboxylase (cODC) degron fused to the human sodium iodide symporter (NIS) gene.

**Figure 2:**
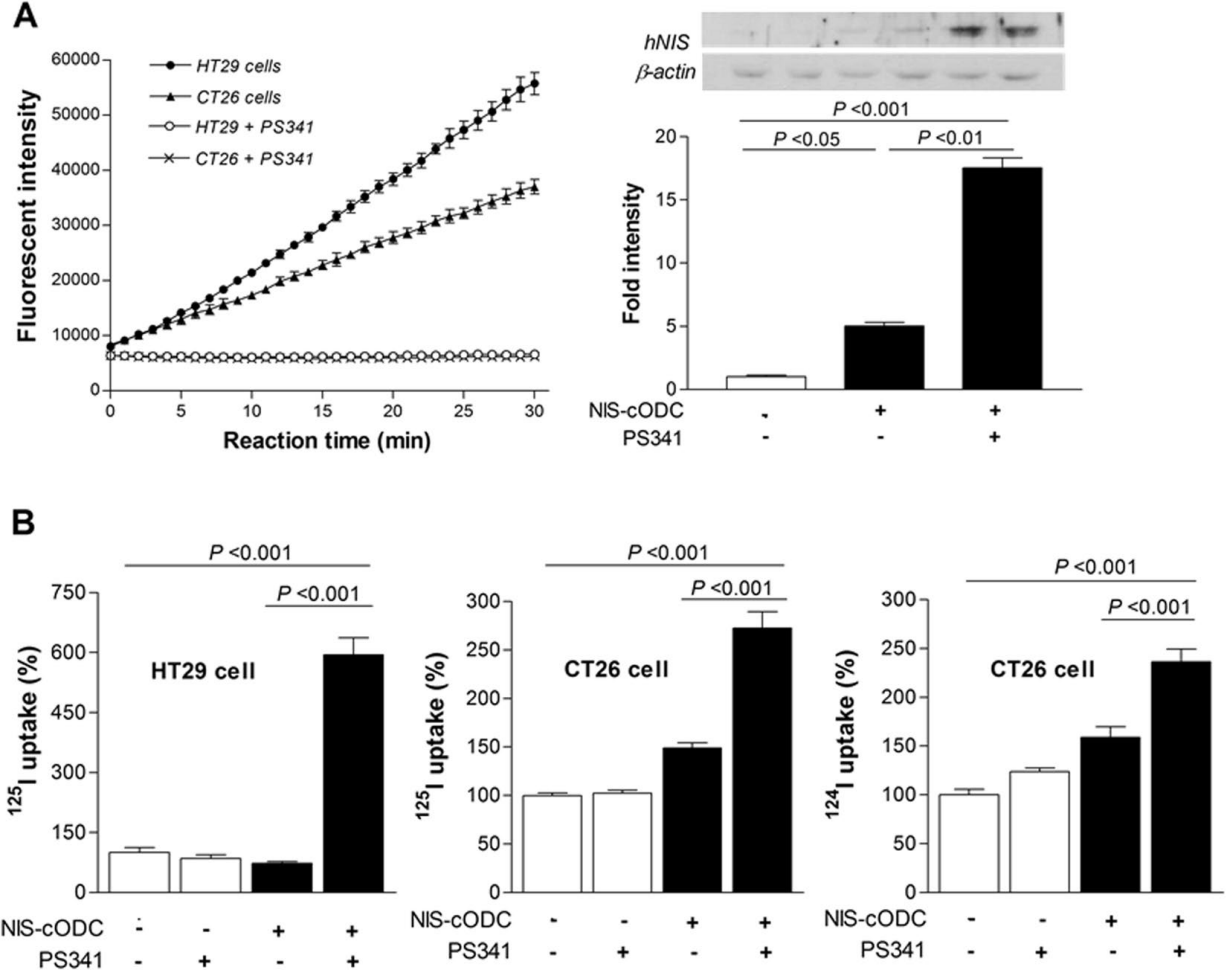
Cancer cells transiently expressing NIS-cODC. (**A**) Bortezomib (PS341) at 50 nM abrogated proteasome activity in CT26 and HT29 colon cancer cells transiently expressing NIS-cODC (left). Data are mean ± standard deviation (SD) of fluorescent intensity of quadruplicate samples. Western blots for NIS and β-actin (loading control) in CT26 cells at day 3 of NIS-cODC transfection (right). Blots were cropped with single blot parts separated by space. For full length blot pictures, see Supplement Fig. 1. Bars are mean ± SD of duplicate band intensities normalized to β-actin bands expressed as fold of controls. (**B**) HT29 cell uptake of ^125^I (left) and CT26 cell uptake of ^125^I (middle) and ^124^I (right) at day 3 of NIS-cODC transfection after 16 h treatment with bortezomib or vehicle. Bars are mean ± SD of % uptake of triplicate samples obtained from a single experiment representative of 2 separate experiments.

Figure 1 illustrates our pQCXIN retroviral expression vector in which the carboxyl terminus 37 amino acids of the murine cODC degron was fused to the NIS gene (NIS-cODC). The vector was first tested by transient transfection in CT26 and HT29 colon cancer cells. Proteasome activity of these cells was completely abrogated by treatment with bortezomib (Fig. 2A). Transfected CT26 cells showed very low NIS expression at baseline, supporting the rapid degradation of NIS-cODC. However, 16 h inhibition of proteasome activity with 4 μM bortezomib induced a marked increase of NIS accumulation that was 17.5 ± 1.1 fold higher than non-transfected cells (Fig. 2A). Furthermore, whereas NIS-cODC transfected cells displayed no increase or only mild increases in radioiodine uptake at baseline, bortezomib stimulated ^125^I uptake to 594.8 ± 73.5% of control level for HT29 cells, and ^125^I and ^124^I uptake to 272.4 ± 29.4% and 236.3 ± 22.8% for CT26 cells (Fig. 2B). Lower baseline uptake level for HT29 compared to CT26 cells suggesting lower leakiness of expression might be explained by the 66.5 ± 1.4% greater proteasome activity for HT29 compared to CT26 cells (Fig. 2A).

### NIS function in stably expressing CT26/NIS-cODC cells treated by bortezomib

**Figure 3:**
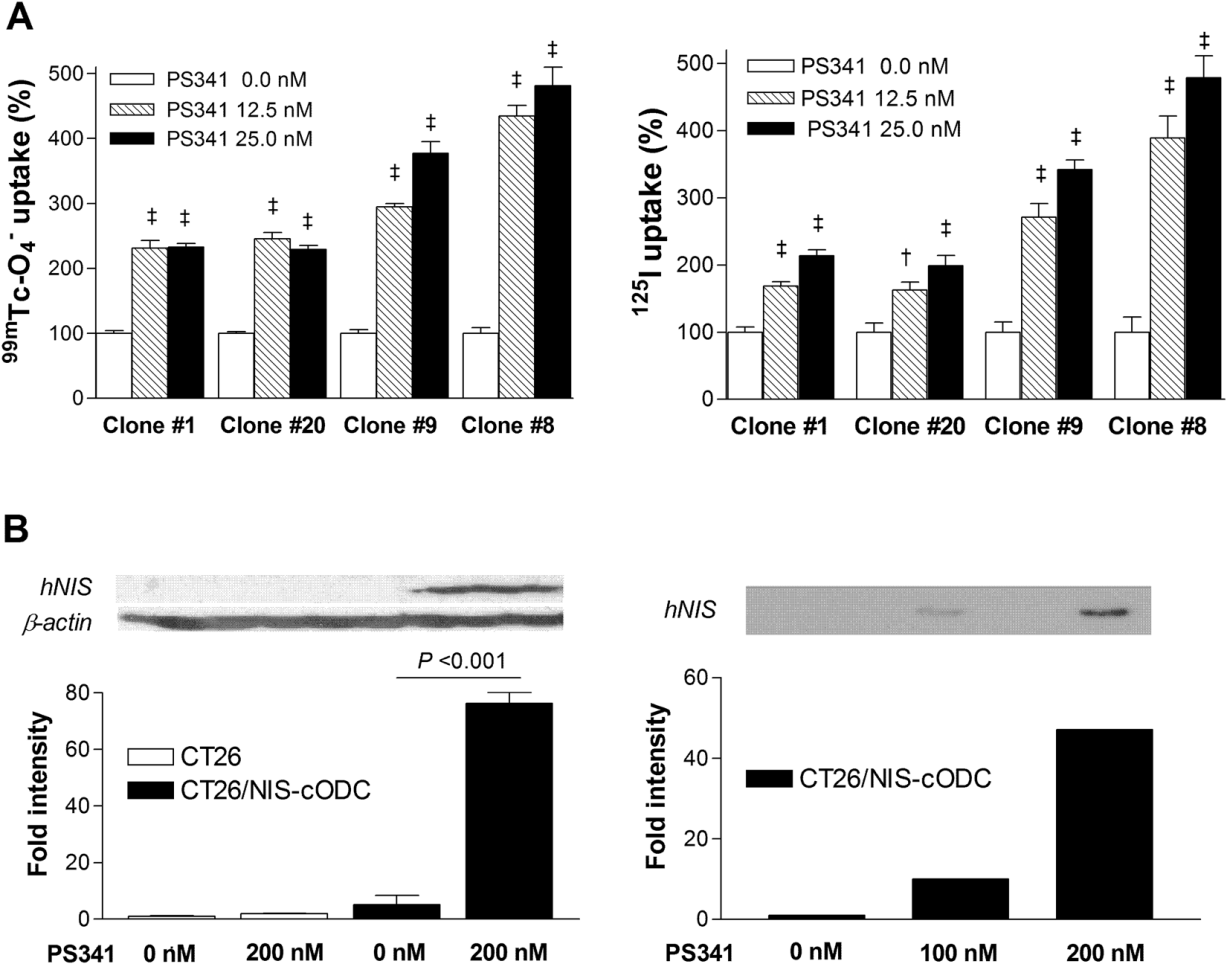
CT26 cells stably expressing NIS-cODC. (**A**) Effects of 48 h exposure to 12.5 or 25 nM bortezomib on uptake of ^99m^Tc-0_4_^−^ (left) and ^125^I (right) on four different stable expressing CT26/NIS-cODC clones. Bars are mean ± SD of % uptake of quadruplicate samples. ^‡^*P* < 0.001, ^†^*P* < 0.005, compared to controls. (**B**) Western blots for NIS and β-actin in CT26 and CT26/NIS-cODC cells using whole cell lysates (left). Cropped blot parts are separated by space. For full length blot pictures, see Supplement Fig. 2. NIS in CT26/NIS-cODC cells using membrane protein (right). Blots were cropped to show representative blots. For full length blot pictures, see Supplement Fig. 3. Bortezomib treatment was for 36 h. Bars on the left side are mean ± SD of duplicate band intensities normalized to β-actin bands expressed as fold of controls.

We next prepared CT26 cells stably expressing NIS-cODC (CT26/NIS-cODC cells) through clonal selection. Exposure of four different CT26/NIS-cODC clones for 48 h with bortezomib caused dose-dependent increases of ^99m^Tc-O_4_^−^ and ^125^I uptake in a highly similar pattern (Fig. 3A). ^99m^Tc-O_4_^−^ and ^125^I uptake reached 481.3 ± 57.0% and 478.8 ± 64.9% of untreated controls, respectively, by 25 nM bortezomib. Furthermore, 36 h treatment of CT26/NIS-cODC cells (clone #9) induced a 76.3 ± 5.5-fold increase of total NIS protein by 200 nM bortezomib, and 10- and 47-fold increases of membrane-localized NIS protein by 100 and 200 nM bortezomib, respectively (Fig. 3B).

**Figure 4:**
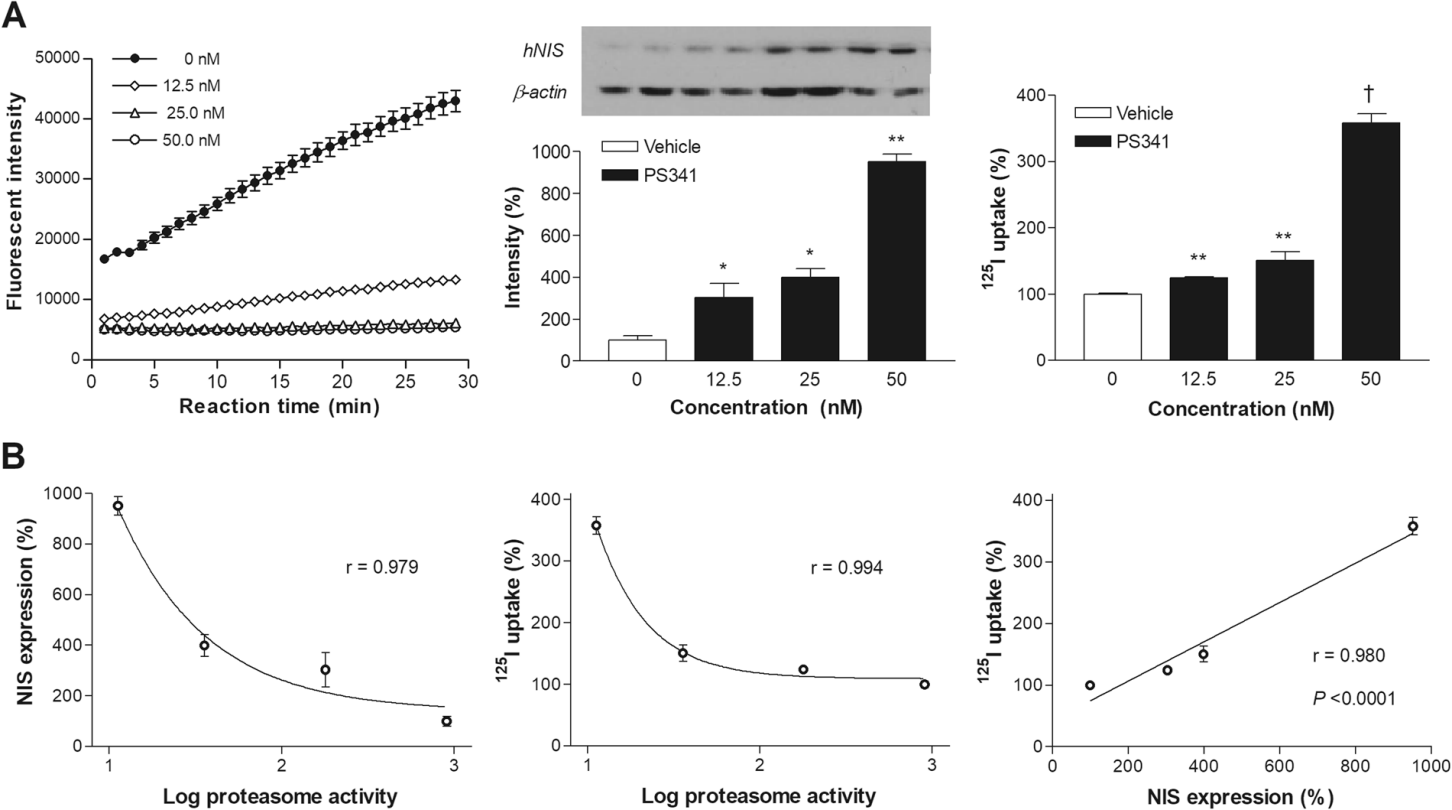
Relation of NIS expression and radioiodine uptake to proteasome activity. (**A**) Proteasome activity (left), NIS expression (middle) and ^125^I uptake (right) in CT26/NIS-cODC cells exposed for 16 h to graded doses of bortezomib. Blots are cropped for representative blots and quantified as mean ± SD in fold of controls of duplicate band intensities normalized to β-actin bands. For full length blot pictures, see Supplement Fig. 4. Bars on the right are mean ± SD of % uptake of triplicate samples. ^‡^*P* < 0.001, **P < 0.01, **P < 0.05, compared to controls. (**B**) Correlations between NIS expression (left) or ^125^I uptake (middle) and log proteasome activity, and between NIS expression and ^125^I uptake (right). Proteasome activity is measured as rate of increase in fluorescent intensity (arbitrary units). Correlation coefficients (r) and *P* values are from regression analysis.

Using CT26/NIS-cODC cells (clone #9), we compared the *in-vitro* activity of the PET reporter system to proteasome activity during treatment with graded doses of PS341. The results showed that proteasome activity was substantially reduced to 24.8% of untreated level by 16 h treatment with 12.5 nM of PS341, and almost completely inhibited by 25 nM or 50 nM (3.1% and 1.2% of untreated level, respectively). Under these conditions, NIS protein level gradually increased to 303.4 ± 68.4%, 398.9 ± 42.4%, and 951.7 ± 36.4% of untreated control level. Similarly, ^125^I uptake level gradually increased to 124.1 ± 2.4%, 150.6 ± 13.2%, and 357.9 ± 14.1% of untreated control level (Fig. 4A). Correlation analysis showed that NIS expression and ^125^I uptake increased exponentially as log proteasome activity decreased. Furthermore, ^125^I uptake level showed a close linear relation to NIS expression level (r = 0.98; P < 0.0001; Fig. 4B).

### Effects of bortezomib on NIS substrate uptake and survival of CT26/NIS-cODC cells

**Figure 5:**
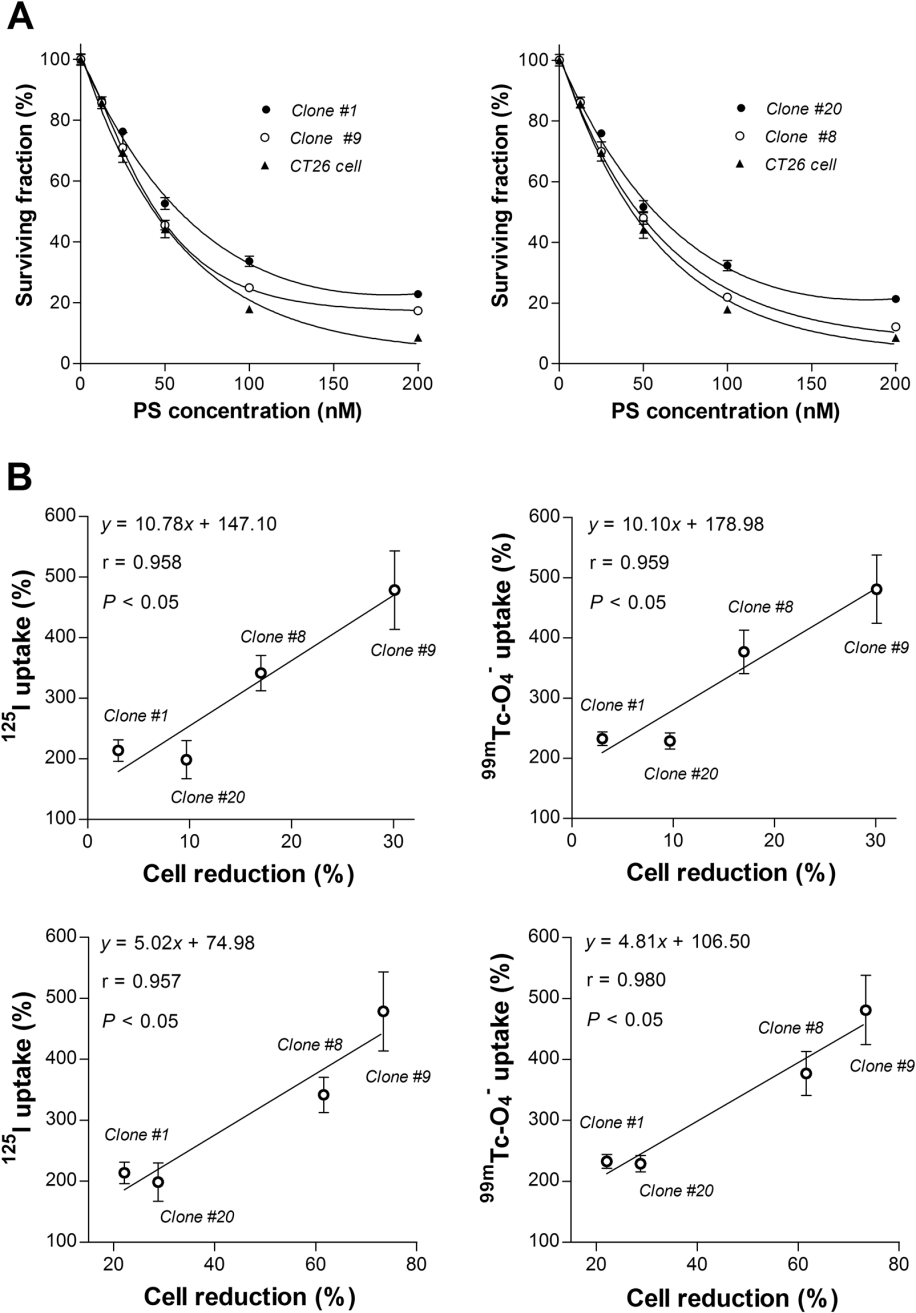
Treatment response of CT26/NIS-cODC clones and relation to NIS transport. (**A**) Surviving fraction of clone #1 and #9 (left) or clone #8 and #20 (right) in comparison to CT26 cells after 72 h treatment with graded doses of bortezomib. Bars are mean ± SD of % survival of quadruplicate samples. (**B**) Linear correlation between ^125^I (left) or ^99m^Tc-0_4_^−^ uptake (right) at 48 h of exposure to 25 nM bortezomib and cytotoxic effects of 72 h treatment with 25 nM (top) or 50 nM bortezomib (bottom) in four different stable CT26/NIS-cODC clones. Bars are mean ± SD of % uptake of quadruplicate samples. Correlation coefficients (r) and *P* values are from linear regression analysis of quadruplicate samples for each stable clone.

We next tested the dose-dependent effects of bortezomib on the survival CT26/NIS-cODC and CT26 cells. After 72 h treatment with 50 nM bortezomib, two CT26/NIS-cODC clones appeared to survive slightly better (52.7 ± 2.0 and 51.7 ± 2.0%) than CT26 cells (44.2 ± 2.8%; both *P* < 0.01), but two other clones showed no difference (45.5 ± 1.5% and 48.1 ± 2.0%; Fig. 5A).

Importantly, we compared bortezomib’s effect on NIS substrate transport and its effect on cell survival. In four different stable CT26/NIS-cODC clones, there was a close correlation between ^125^I uptake in cells exposed to 25 nM bortezomib for 48 h and the magnitude of cell loss by treatment with 25 nM (r = 0.958, *P* < 0.05) or 50 nM bortezomib (r = 0.957, *P* < 0.05; Fig. 5B). ^99m^Tc-O_4_^−^ uptake in cells exposed to 25 nM bortezomib for 48 h faithfully recapitulated this close correlation with cell loss by treatment with 25 nM (r = 0.959, *P* < 0.05) or 50 nM bortezomib (r = 0.980, *P* < 0.05; Fig. 5B).

### ^124^I PET imaging quantitatively monitors tumor response to bortezomib treatment

**Figure 6:**
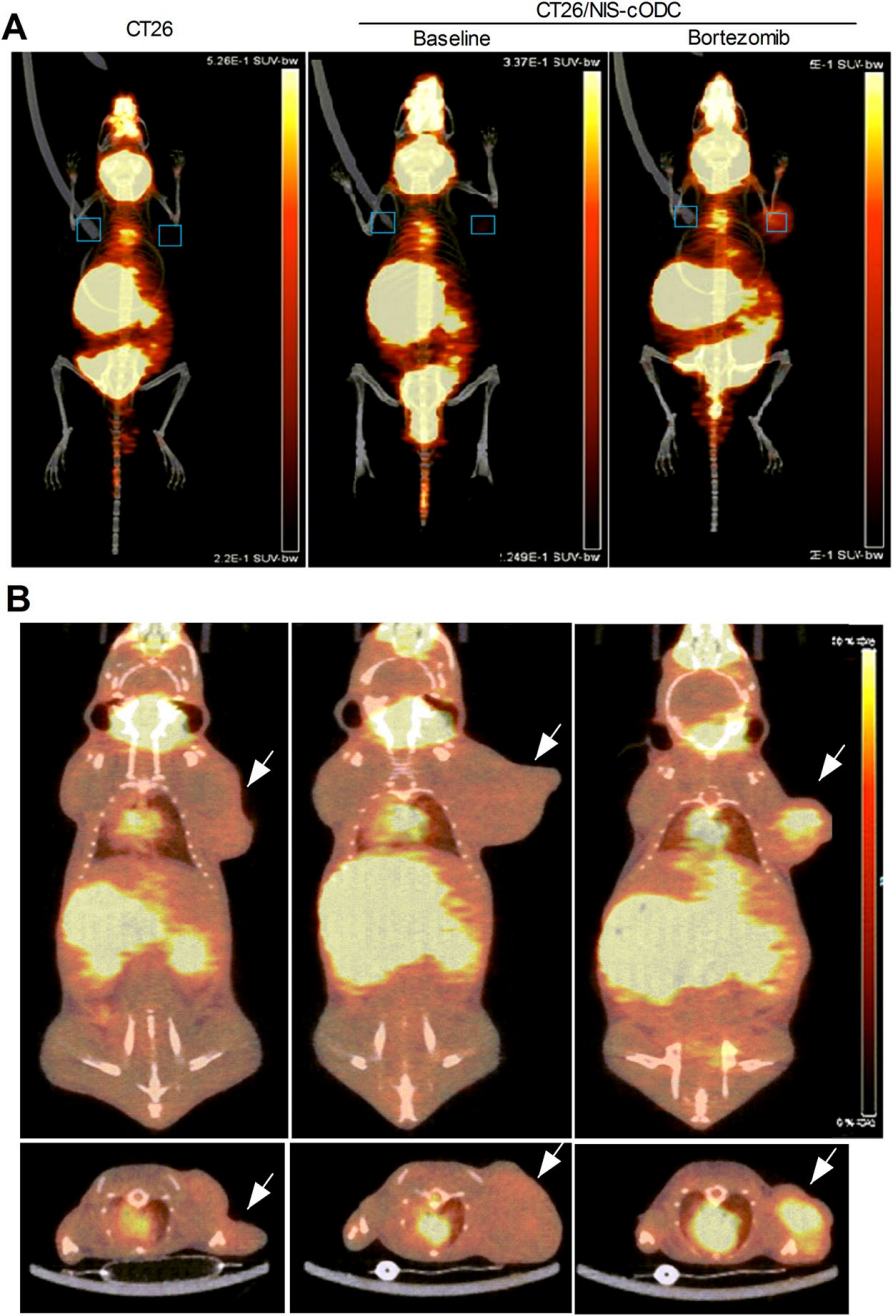
PET/CT imaging of tumor response to Bortezomib treatment. (a,b) *In vivo* maximum intensity projection (MIP) views (**A**), and coronal (**B**, top) and transaxial (b, bottom) tomographs of ^124^I PET/CT images of mice harboring CT26 (left, arrow) or CT26/NIS-cODC tumors (arrows, middle and right). Animals with CT26/NIS-cODC tumors were injected with 1 mg/kg bortezomib (PS341; right) or vehicle (DMSO; middle) at 24 h and 12 h prior to PET imaging. Boxes in MIP views show ROIs placed on tumor and contralateral background regions.

**Figure 7:**
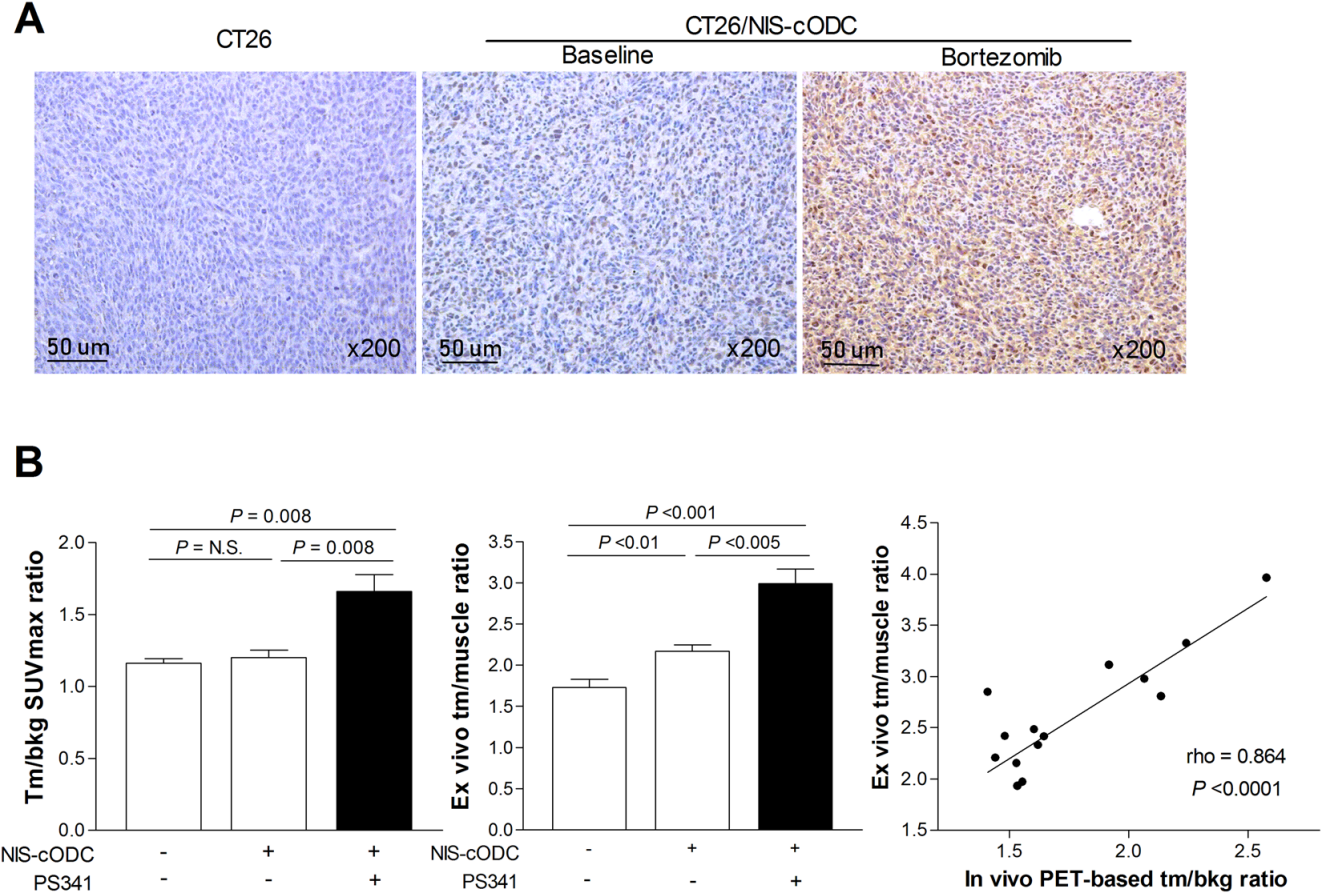
Tumor NIS staining and radioiodine uptake level. (**A**) Immunohistochemistry for NIS expression in tumor tissues extracted after imaging. (**B**) *In vivo* tumor-to-background SUVmax ratios obtained from PET (left), and *ex vivo* tumor-to-muscle (tm/muscle) count ratios (middle). Bars are mean ± SD of values obtained from mice with CT26 tumors (n = 5) and mice with CT26/NIS-cODC tumors treated with vehicle (n = 6) or bortezomib (n = 8). Correlation between *in vivo* and *ex vivo* measured uptake indices with Spearman’s correlation coefficient (rho; right).

To investigate the usefulness of NIS-cODC reporter imaging for monitoring proteasome inhibitor response *in vivo*, we prepared mice harboring subcutaneous CT26 or CT26/NIS-cODC tumors. PET/CT imaging showed that, without bortezomib treatment, neither type of tumors took up significant amounts of ^124^I (Fig. 6A,B). However, after treatment with just 2 doses of bortezomib, CT26/NIS-cODC tumors displayed significantly increased ^124^I uptake (Fig. 6A,B). Immunohistochemistry confirmed high levels of NIS protein in tumors of this group, but very small amounts of NIS in tumors of the other two groups (Fig. 7A). Quantitative PET analysis confirmed greater tumor-to-background SUVmax ratios for bortezomib-treated CT26/NIS-cODC tumors (1.66 ± 0.33) compared to CT26 tumors (1.16 ± 0.07, *P* = 0.008) or CT26/NIS-cODC tumors without treatment (1.20 ± 0.13, *P* = 0.008; Fig. 7B). SUVmean ratios of uptake were also significantly greater for bortezomib-treated CT26/NIS-cODC tumors (2.02 ± 0.53) compared to CT26 tumors (1.32 ± 0.14, *P* = 0.009) or CT26/NIS-cODC tumors without treatment (1.32 ± 0.11, *P* = 0.012). *Ex vivo* measured tumor-to-muscle count ratios of extracted tissue reiterated these results (2.99 ± 0.49 vs. 1.73 ± 0.22 and 2.17 ± 0.19, respectively; Fig. 7B). Correlation analysis verified a high correlation between PET image-based SUVmax ratios and *ex vivo* measured tissue count ratios (rho = 0.864, *P* < 0.0001; Fig. 7B).

## Discussion

Construction of reporters that contain the cODC sequence provides a unique opportunity to monitor proteasome activity. Although cODC-fused fluorescent reporters are useful for identifying cancer cells with poor proteasome activity *in vitro*, poor signal penetrance limits their value for imaging living bodies. Here, we introduce a noninvasive PET reporter system based on cODC-fused NIS that allows quantitative imaging of cancer cells with suppressed proteasome activity *in vivo*.

The cODC consists of a 37-residue terminal-sequence that is recognized and rapidly degraded by the 26S proteasome. Because this occurs in a ubiquitin-independent manner, no posttranslational modifications are required for substrate preparation. Hence, cODC-tagged constructs readily act as substrates to probe proteasome-mediated degradation *in vitro* as well as *in vivo*. This degradation tag functions autonomously and can be appended to other proteins for proteasome targeting^9^. The cODC sequence is highly conserved, and tagging with the murine cODC leads to efficient degradation of proteins from various species by mammalian proteasomes^17^.

Although CT26 and HT29 cells are derived from colon tumors, they do not show significant endogenous NIS expression^13,18,19^. In our study, we transduced cells with a retroviral vector driven by the CMV promoter, which results in constitutive expression cODC-NIS, uninfluenced by the environment. In our study, CT26 and HT29 cells showed complete abrogation of proteasome activity in response to bortezomib treatment. In accordance with the rapid degradation of cODC-fused proteins under steady-state conditions, cells transiently transduced with NIS-cODC showed very low NIS expression at baseline. However, the cells displayed a substantial increase of NIS protein when proteasome activity was inhibited by bortezomib. The minimal amount of NIS-cODC protein at baseline allowed reporting of low proteasome activity with increases of NIS-mediated uptake of radioiodine and radio-technetium substrates.

Treatment of stably transfected CT26/NIS-cODC clones to bortezomib caused dose-dependent increases of ^99m^Tc-O_4_^−^ and ^125^I uptakes. In clones 1 and 20, ^125^I uptake was higher by treatment with 25 nM of PS341 compared to 12.5 nM, but ^99m^Tc-O_4_^−^ uptake was not. Although the cause for this finding is not clear, it is noteworthy that these 2 clones had apparently lower NIScODC transfection efficiency compared to clones 9 and 8. ^99m^Tc-O_4_^−^ has been reported to rapidly efflux out of cancer cells transfected with the NIS gene^20^. It might therefore be possible that ^99m^Tc-O_4_^−^ efflux in these cells is more rapid than that of radioiodine, causing less apparent differences in ^99m^Tc-O_4_^−^ accumulation according to NIS expression in cancer cells with low transfection efficiency.

The crucial role proteasomes play in degrading regulatory proteins that suppress cancer cell proliferation has led to clinical trials of proteasome inhibitors for cancer treatment. The ability to monitor proteasome activity of tumors *in vivo* can contribute to the development of new drugs that are more effective and apply to a broader range of malignancies^2,6,7^. We investigated this issue in CT26 cells engineered to stably express NIS-cODC. Our results showed that graded concentrations of bortezomib resulted in substantial and dose-dependent reductions of cell number by bortezomib for both CT26 and CT26/NIS-cODC cells. In contrast to a previous study where stable ZsGreen-cODC expression was found to be stressful for cancer cells^11^, we found that NIS-cODC expression by itself did not exert cellular stress. Importantly, the amount of increase of NIS substrate transport in bortezomib-treated CT26/NIS-cODC cells closely correlated to the magnitude of suppression in cell survival. This finding supports the possibility that the level of stimulated radioiodine uptake might help monitor cancer cell response to proteasome inhibitor treatment.

Finally, we investigated the ability of our reporter system to image inhibited proteasome activity in tumors of living mice. For this purpose, CT26 cells conveniently produced a syngeneic tumor model in wild type BALB/c mice. PET/CT imaging of CT26/NIS-cODC tumors revealed significantly increased radioiodine uptake after only 2 doses of bortezomib. This drug is considered a potent and specific proteasome inhibitor compared to the less specific and weaker predecessors, making it suitable for *in vivo* application^21^. Bortezomib is the first-in-class therapeutic proteasome inhibitor approved for human use, and several clinical studies have established its efficacy in multiple types of refractory cancers^22^. In our experiments, CT26/NIS-cODC tumors of bortezomib-treated animals displayed clearly increased radioiodine uptake on PET and showed strong NIS expression on tissue staining, which demonstrates the capacity of our reporter to noninvasively image tumor response to proteasome inhibition.

In clinical trials, bortezomib is administered at doses of up to 2 mg/m^2^ for a cumulative dose of 40 mg/m^2 ^^23^. Assuming a body-mass index of 25 kg/m^2^, these doses would amount to approximately 0.08 and 1.6 mg/kg, respectively. Extrapolation of human doses to mice cannot be done based on body weight alone, because species-dependent biochemical and functional systems alter pharmacokinetics. Small animals require larger drug dose compared with humans on a weight basis, and for mice, a multiplication correction factor of 12.3 is recommended^24^. In our case, this yields a single dose of approximately 1 mg/kg, which is the dose that we used. In a previous study, Momose *et al*. imaged mice harboring HEK293 tumors that stably expressed ZsGreen-cODC after injecting a single dose of bortezomib^12^. In their study, tumor fluorescence was greatest with 4 mg/kg bortezomib and weak with 1 mg/kg. Therefore, it is likely that higher doses of bortezomib would have resulted in higher levels of tumor radioiodine uptake.

In our study, radioiodine uptake assessed *in vivo* by PET was highly accurate in comparison to uptake confirmed from extracted tissues. Whereas optical imaging of fluorescent signals is limited by depth of light penetration and scatter, gamma rays generated from positron annihilation have high tissue penetrance. Hence, these issues are not limiting for PET-based reporter imaging^25^. Therefore, our NIS reporter PET system may provide highly accurate quantitative measurements and is expected to work just as well for deep seated orthotopic tumor models as in subcutaneous tumor models. Together, our results demonstrate the potential usefulness of NIS-cODC reporter PET for noninvasive monitoring of tumor response to proteasome inhibitors.

A limitation of this study is that we did not compare radioiodine uptake level stimulated by bortezomib to actual antitumor effects. Although this experiment was beyond the scope of the present study, which was to investigate the capacity of our reporter PET system to image proteasome inhibition, the issue deserves exploration in future studies.

In conclusion, we have developed a novel reporter PET technology based on the NIS gene fused with the cODC degron, which allows robust monitoring of suppressed cancer cell proteasome activity in response to treatment. Accumulation of NIS-cODC protein by low proteasome activity led to increased tumor radioiodine uptake that was noninvasively imaged by PET in a quantitative manner. This technique may therefore be useful for noninvasive monitoring of tumor response to proteasome inhibitors, which could facilitate new drug development.

## Materials and Methods

### Construction of the NIS-cODC expression cassette and retroviral vector

The cODC sequence directs the recognition site of the proteasome. A retroviral expression vector containing the cODC degron fused to the ZsGreen gene (pQCXIN-ZsGreen-cODC) was a kind gift from Dr. Frank Pajonk (David Geffen School of Medicine at University of California, LA). We removed the ZsGreen gene sequence from this vector by digestion with *Bam* HI and *Eco* RI restriction enzymes (Takara, CA). The human NIS gene sequence cut out with identical restriction enzymes was then cloned into the vector to produce pQCXIN-NIS-cODC, which expresses the NIS-cODC fusion protein. The wild type human NIS that we used efficiently transports radioiodine in various *in vitro* and *in vivo* systems^13,14,16^. This gene sequence is comprised of 2490 nucleotides that encode a protein of 643 amino acids as previously described^26^. pQCXIN-NIS-cODC was transfected into PT67 packaging cells (Clontech, CA) in high-glucose DMEM media (Lonza, Basel, Switzerland) to produce retroviral vectors.

### Cell culture and transient transfection

CT26 mouse colon cancer cells and HT29 human colon cancer cells from the American Type Culture Collection were maintained in RPMI (Lonza) supplemented with 10% fetal bovine serum (Serana, Germany) and 1% penicillin/streptomycin (Gibco Laboratories, Gaithersburg, MD) at 37 °C and 5% CO_2_ in a humidified atmosphere. Cells were sub-cultured twice a week. To prepare transient transfectants, 5 × 10^4^ cells were seeded into 24-well plates and transduced with pQCXIN-NIS-cODC or empty pQCXIN plasmids (control) by adding 2 μg DNA per well mixed with 2 μl lipofectamine LTX (Invitrogen, Carlsbad, CA) in opti-MEM (Gibco). Proteasome inhibition was performed by adding bortezomib (PS341; Calbiochem, MA) to the culture media.

### Stably expressing cell lines

Stably-expressing CT26 cells were prepared because, unlike HT29 tumors that require immune-deficient mice, CT26 tumors are conveniently generated in wild-type mice. CT26 cells were infected with retroviral particles and selected 72 h later under 200 μg/ml geneticin (Gibco). Single-cell clones expressing NIS-cODC (CT26/NIS-ODC cells) or ZsGreen-cODC (CT26/ZsGreen-cODC cells) were isolated by plating dilute suspensions, and surviving clones were picked up, amplified in media containing geneticin, and stored in liquid nitrogen.

### Immunoblotting for NIS proteins

Cells were lysed with cold protein extraction solution (PRO-PREP; Intron, Korea) containing a protease inhibitor cocktail (Sigma Aldrich, St. Louis, MO). Membrane protein was also prepared to assess membrane localized NIS. For this, cells were scrapped and homogenized in lysis buffer containing sucrose (0.0856 g/mL), 10 mM *N*-(2-hydroxyethyl)piperazine-*N’*-(2-ethanesulfonic acid), 1 mM ethylenediaminetetraacetic acid, 10 μg/mL aprotinin, and 1 mM phenylmethylsulfonylfluoride. Following removal of cell debris, the supernatant was incubated with 0.1 M Na_2_CO_3_ at 4 °C for 1 h. The samples were filled with buffer containing sucrose (0.0856 g/mL), 10 mM HEPES, and 1 mM MgCl_2_ and then centrifuged at 42,000 rpm for 1 h at 4 °C. After centrifugation, the membrane fraction pellet was suspended in phosphate buffered saline (PBS).

Cell lysate (30 μg) or membrane fraction protein (10 μg) were separated by electrophoresis on a 10% sodium dodecyl sulfate polyacrylamide gel, followed by transfer to a polyvinylidene difluoride membrane (Bio-RAD, CA). The membrane was blocked with 5% nonfat milk in Tris-buffered saline and polysorbate-20 for 1 h at room temperature and incubated overnight at 4 °C with primary antibodies against NIS or β-actin (Santa Cruz Biotechnology, TX). Membranes were then incubated at room temperature for 1 h with secondary anti-rabbit IgG antibody for NIS and anti-mouse IgG antibody for β-actin (Cell Signaling, MA). Immune reactive proteins were detected by chemiluminescence, and band intensities were quantified on a GS-800 densitometer using Quantity One software (Bio-Rad Laboratories).

### Cellular radioiodine and radio-technetium uptake measurements

Cells were incubated for 1 h with 74 kBq ^125^I (Perkin Elmer, MA), 110 kBq ^124^I, or 740 kBq ^99m^Tc-O_4_^−^ added to the culture media in 5% CO_2_ at 37 °C. Cells were rapidly washed twice with cold PBS, lysed with 0.1 N NaOH, and measured for cell-bound radioactivity on a high energy γ-counter (Perkin-Elmer) for ^124^I or ^99m^Tc-O_4_^−^ and γ-counter (Wallac) for ^125^I. Uptake levels were normalized to protein content for each sample.

### Proteasome activity assay

To measure proteasome activity, 1.2 × 10^6^ cells in 6-well plates were washed and collected by scraping with 0.5% NP-40 in distilled water. After incubation for 30 min at −20 °C and centrifugation at 13,000 rpm for 10 min at 4 °C, supernatants were measured for proteasome activity with a fluorometric assay kit (Bio Vision, CA). Briefly, cell extracts were mixed with 100 μl assay buffer and 1 μl Suc-LLVY-7-amino-4-methylcoumarin (Suc-LLVY-AMC) substrate. Fluorescence was measured at 350 nm excitation and 440 nm emission wavelengths in a microplate reader at 37 °C for 30 min. Proteasome activity was measured as rate increase of fluorescent signals from Suc-LLVY-AMC degradation.

### Sulforhodamine B (SRB) survival assay

Surviving cell content was measured by SRB assays. Briefly, cells seeded overnight on a 96-well plate were treated for the indicated durations of time, and cell monolayers were fixed with 10% (wt/vol) trichloroacetic acid at 4 °C. After cells were stained with SRB dye (Sigma-Aldrich) for 30 min, excess dye was removed by repeated washing with 1% (v/v) acetic acid. Protein-bound dye was finally dissolved in 10 mM Tris base solution and subject to spectrophotometric measurement of absorbance at 510 nm using a microplate reader.

### *In vivo*^124^I PET imaging and *ex vivo* tumor uptake

Animal experiments were performed in accordance with the National Institutes of Health Guide for Care and Use of Laboratory Animals, and approved by the Institutional Animal Care and Use Committee of Samsung Medical Center. Three groups of tumor-bearing animals were prepared in 8-week-old wild type male BALB/c mice by subcutaneous injection of 1 × 10^8^ cancer cells into the right shoulder region. The control group had tumors formed by non-transduced CT26 cells (n = 5). The second and third groups had CT26/NIS-cODC tumors and were intraperitoneally injected with DMSO vehicle (n = 6) or 1 mg/kg bortezomib (n = 8), respectively, at 12 and 24 h prior to PET imaging. When tumor size reached approximately 1.5 cm in diameter, animals were injected with 4 MBq of ^124^I through the tail vein. Imaging was performed 1 h later under isoflurane anesthesia, using a small-animal PET/CT scanner (Inveon; Siemens, Germany) without respiratory gating. PET imaging was followed by non-enhanced CT without attenuation correction. Care was taken to maintain an environment of 23–25 °C.

A square ROI was placed on the tumor region with highest activity on coronal PET, and a mirrored ROI was placed on the contralateral shoulder as background. Tumor-to-background ratios of mean and maximum standardized uptake values (SUVmean and SUVmax) were used as indices of uptake. Immediately after PET imaging, animals were sacrificed by cervical dislocation. Tumor and background tissues were extracted, weighed, and measured for ^124^I radioactivity on a high-energy γ-counter. *Ex vivo* measurements were expressed as weight-corrected tumor-to-muscle count ratios.

### Immunohistochemistry staining of tissue sections for NIS expression

After micro-sectioning formalin-fixed and paraffin-embedded tissues at 4 μm thickness, heat-induced antigen retrieval was performed for 3 min in pH 9.0 EDTA buffer (Dako). Sectioned slides were then incubated overnight with an anti–human NIS rabbit polyclonal antibody (1:100 dilution; Santa Cruz Biotechnology) at 4 °C. This was followed by incubation with HRP-labeled polymer-conjugated secondary antibodies against rabbit IgG (Dako, CA) for 30 min at room temperature. A color reaction was induced using the ready-to-use 3,3′-diaminobenzidine substrate chromogen solution (Dako) for 5 min, followed by washing with distilled water. Sections were lightly counterstained with hematoxylin for 30 seconds before dehydration and mounting.

### Statistical analysis

Data are presented as mean ± standard deviation (SD). Significance of difference between groups was analyzed by two-tailed unpaired Student’s *t*-tests for two groups and ANOVA with Tukey’s post-hoc tests for three or more groups. Correlation was evaluated by linear regression or Spearman’s rank-order analysis. *P* values < 0.05 were considered statistically significant.

### Data Availability

All data generated or analyzed during this study are included in this published article.

## Electronic supplementary material

Supplementary Information
