# Supplementary material for: Reporter PET Images Bortezomib Treatment-Mediated Suppression of Cancer Cell Proteasome Activity
Source: Sci Rep. 2018 Aug 16;8:12290. doi: 10.1038/s41598-018-29642-w (PMC6095884; doi:10.1038/s41598-018-29642-w)
Supplement: Supplementary file 1 — Supplementary Information [file 41598_2018_29642_MOESM1_ESM.docx]

**Supplementary information**

**Reporter PET Images Bortezomib Treatment-Mediated**

**Suppression of Cancer Cell Proteasome Activity**

Jin Hee Lee*^1,2^*, Kyung-Ho Jung*^1,2^*, Cung Hoa Thien Quach*^1^*, Jin Won Park*^1,2^*,

Seung Hwan Moon*^1^*, Young Seok Cho*^1^*, Kyung-Han Lee*^1,2^*^,*^

^1^Department of Nuclear Medicine, Samsung Medical Center Seoul, Korea

^2^Department of Health Sciences and Technology, SAIHST, Sungkyunkwan University School of Medicine, Seoul, Korea

*Corresponding Author (For reprints): Kyung-Han Lee, MD, PhD.

Department of Nuclear Medicine, Samsung Medical Center,

50 Ilwon-dong, Gangnam-gu, Seoul, Korea.

Tel : 82-2-3410-2630; Fax : 82-2-3410-2639; [khnm.lee@samsung.com](mailto:khnm.lee@samsung.com)

**
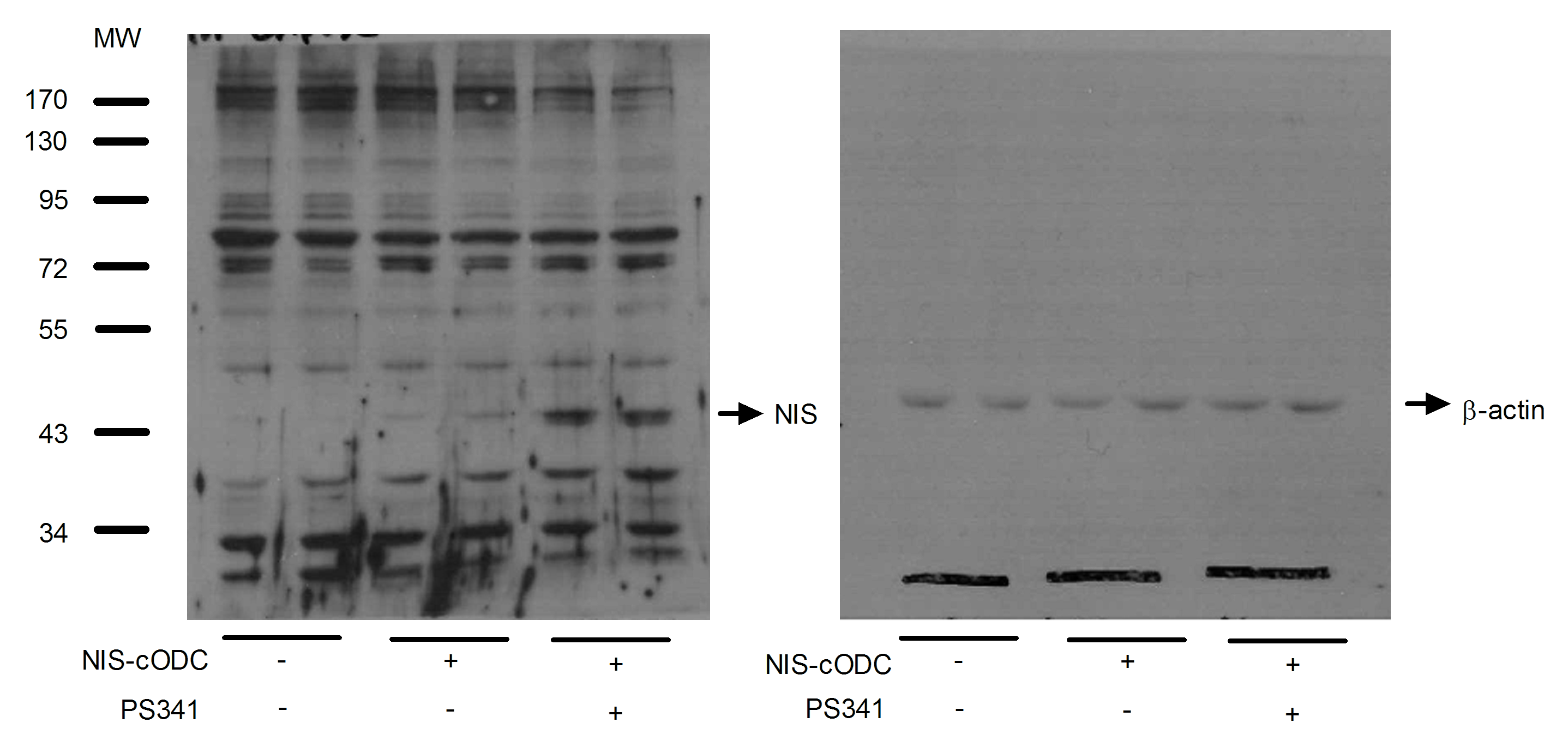
**

Supplemental Figure 1: Full length blots from Figure 2A. Western blots of protein from cell lysate for detection of NIS (left) and β-actin (right; after stripping of NIS antibodies). MW, molecular weight.

**
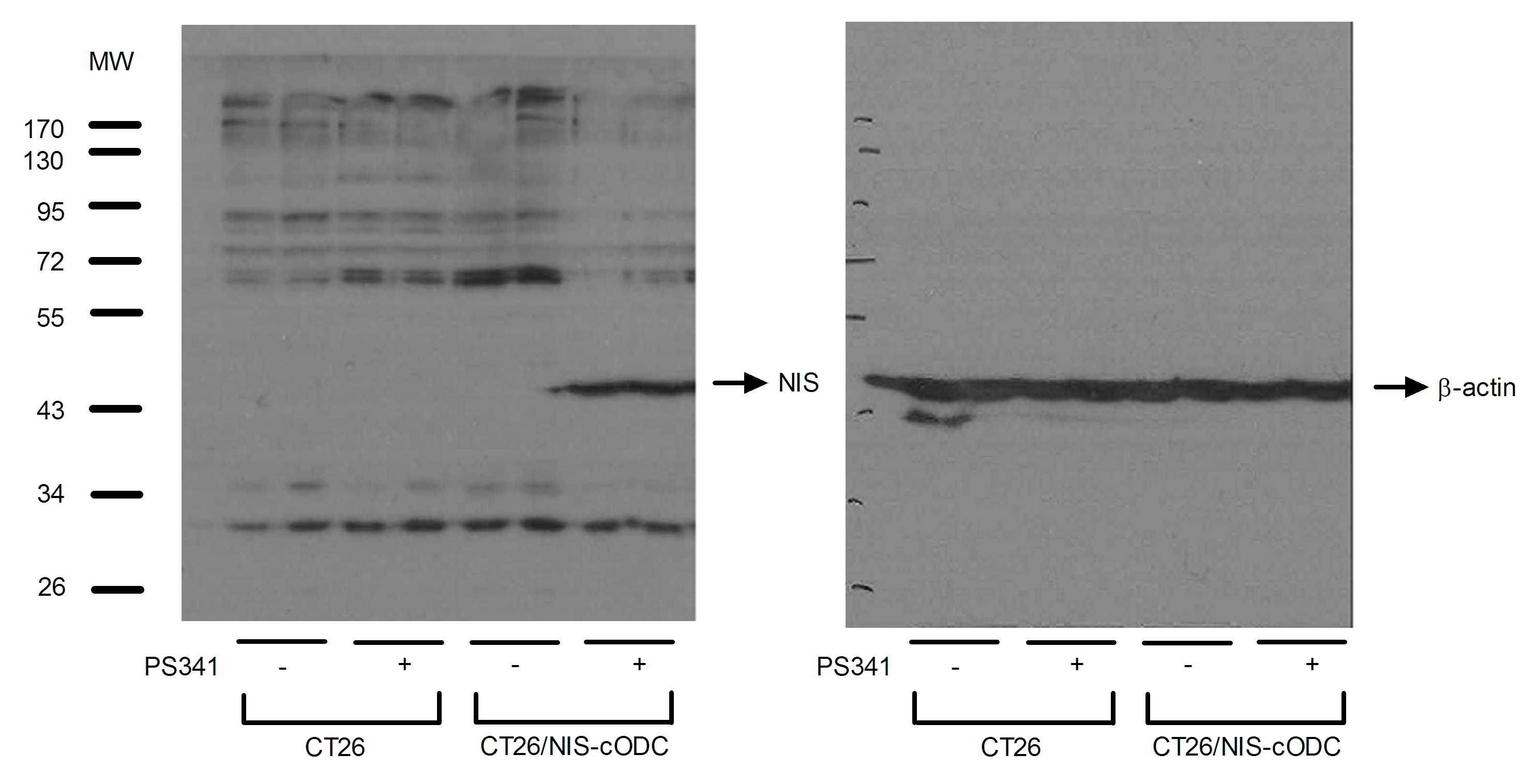
**

Supplemental Figure 2: Full length blots from Fig. 3B (left). Western blots of protein from cell lysate for detection of NIS (left) and β-actin (right; after stripping of NIS antibodies). MW, molecular weight.

**
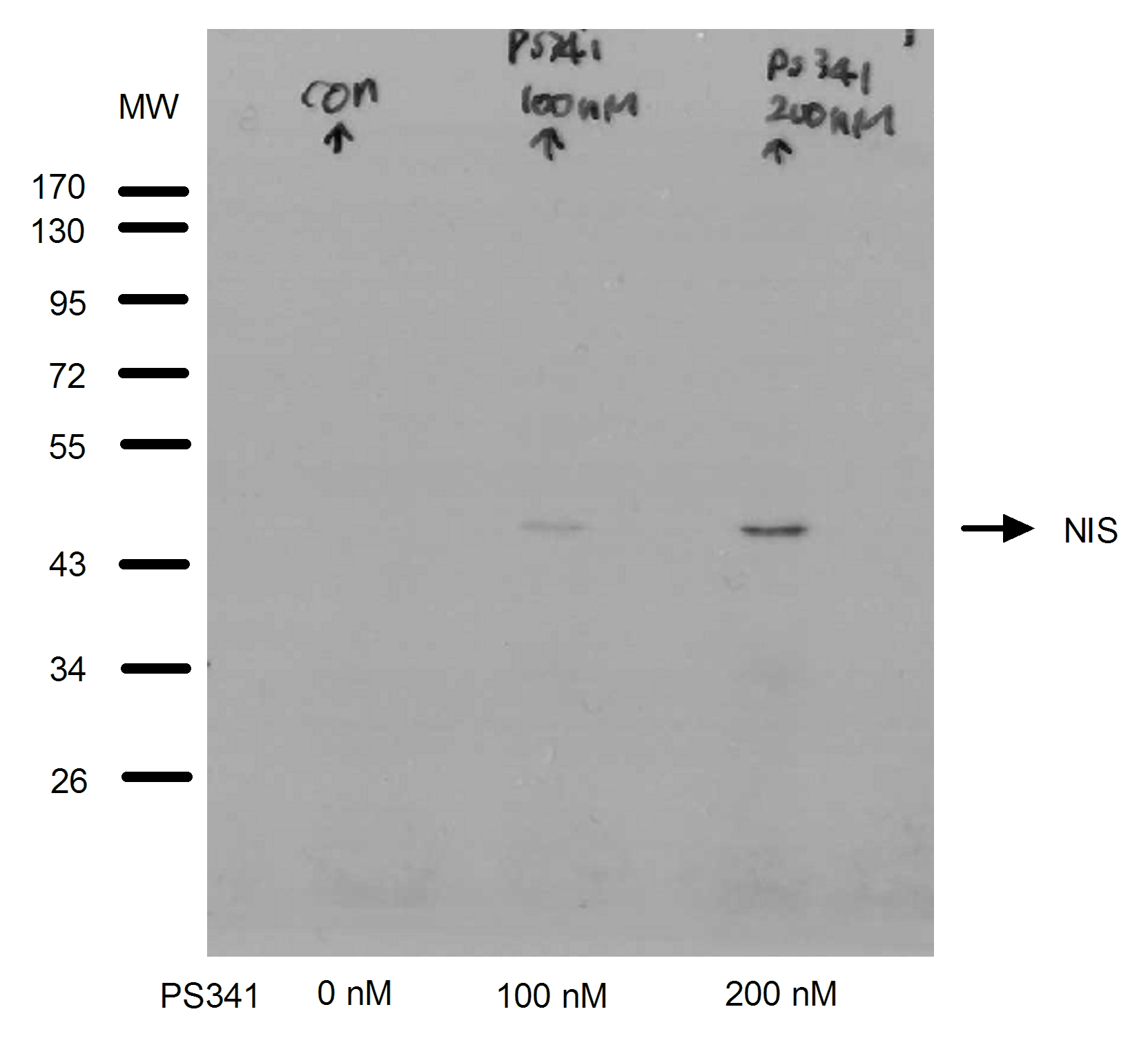
**

Supplemental Figure 3: Full length blot from Fig. 3b (right). Western blot of cell membrane fraction protein for detection of NIS. MW, molecular weight.


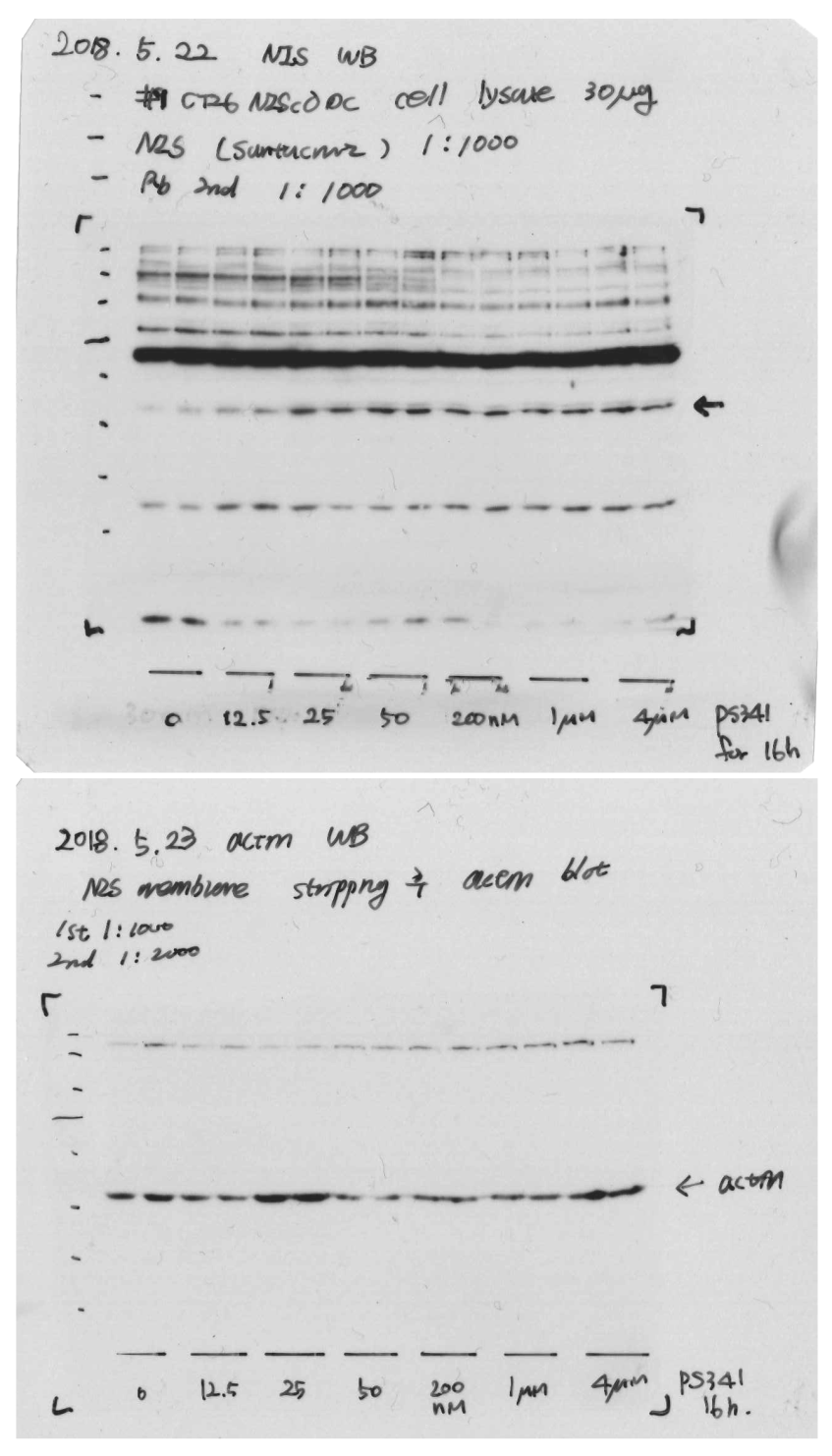


Supplemental Figure 4: Full length blot from Fig. 4a (middle). Western blots of protein from cell lysate for detection of NIS (top, arrow) and β-actin (bottom; arrow, after stripping of NIS antibodies).
